# Antifungal susceptibility of invasive yeast isolates in Italy: the GISIA3 study in critically ill patients

**DOI:** 10.1186/1471-2334-11-130

**Published:** 2011-05-17

**Authors:** Giulia Morace, Elisa Borghi, Roberta Iatta, Gerardino Amato, Stefano Andreoni, Gioconda Brigante, Claudio Farina, Giuliana Lo Cascio, Gianluigi Lombardi, Ester Manso, Michele Mussap, Patrizia Pecile, Roberto Rigoli, Elisabetta Tangorra, Maria Valmarin, Maria Teresa Montagna

**Affiliations:** 1 Department of Public Health - Microbiology - Virology, Università degli Studi di Milano, Milan, Italy; 2 Department of Biomedical Science and Human Oncology, Hygiene Section, Università degli Studi di Bari, Bari, Italy; 3 Laboratory of Clinical Pathology and Microbiology, Ospedale Cardarelli, Naples, Italy; 4 Laboratory of Microbiology and Virology, Ospedale Maggiore della Carità, Novara, Italy; 5 Laboratory of Microbiology, Ospedale Circolo, Varese, Italy; 6 Laboratory of Microbiology and Virology, Ospedale San Carlo Borromeo, Milan, Italy; 7 Laboratory of Microbiology and Virology, Ospedale GB Rossi, Verona, Italy; 8 Laboratory of Microbiology and Virology, Ospedale Niguarda Ca' Granda, Milan, Italy; 9 Laboratory of Microbiology, Ospedale Torrette, Ancona, Italy; 10 Laboratory of Microbiology, Ospedale San Martino, Genoa, Italy; 11 Laboratory of Microbiology, Ospedale Careggi, Florence, Italy; 12 Laboratory of Microbiology and Virology, Ospedale Ca' Foncello, Treviso, Italy; 13 Department of Haematology and Oncologic Sciences, Università degli Studi di Bologna, Bologna, Italy; 14 Laboratory of Microbiology and Virology, Ospedale Forlanini, Rome, Italy

## Abstract

**Background:**

Yeasts are a common cause of invasive fungal infections in critically ill patients. Antifungal susceptibility testing results of clinically significant fungal strains are of interest to physicians, enabling them to adopt appropriate strategies for empiric and prophylactic therapies. We investigated the antifungal susceptibility of yeasts isolated over a 2-year period from hospitalised patients with invasive yeast infections.

**Methods:**

638 yeasts were isolated from the blood, central venous catheters and sterile fluids of 578 patients on general and surgical intensive care units and surgical wards. Etest strips and Sensititre panels were used to test the susceptibility of the isolates to amphotericin B, anidulafungin, caspofungin, fluconazole, itraconazole, posaconazole and voriconazole in 13 laboratories centres (LC) and two co-ordinating centres (CC). The Clinical and Laboratory Standards Institute (CLSI) reference broth microdilution method was used at the CCs for comparison.

**Results:**

Etest and Sensititre (LC/CC) MIC_90 _values were, respectively: amphotericin B 0.5/0.38, 1/1 mg/L; anidulafungin 2/1.5 and 1/1 mg/L; caspofungin 1/0.75 and 0.5/0.5 mg/L; fluconazole 12/8 and 16/16 mg/L; itraconazole 1/1.5, 0.5/0.5 mg/L; posaconazole 0.5 mg/L and voriconazole 0.25 mg/L for all. The overall MIC_90 _values were influenced by the reduced susceptibility of *Candida parapsilosis *isolates to echinocandins and a reduced or lack of susceptibility of *Candida glabrata *and *Candida krusei *to azoles, in particular fluconazole and itraconazole. Comparison of the LC and CC results showed good Essential Agreement (90.3% for Etest and 92.9% for Sensititre), and even higher Categorical Agreement (93.9% for Etest and 96% for Sensititre); differences were observed according to the species, method, and antifungal drug. No cross-resistance between echinocandins and triazoles was detected.

**Conclusions:**

Our data confirm the different antifungal susceptibility patterns among species, and highlight the need to perform antifungal susceptibility testing of clinically relevant yeasts. With the exception of a few species (e.g. *C. glabrata *for azoles and *C. parapsilosis *for echinocandins), the findings of our study suggest that two of the most widely used commercial methods (Etest and Sensititre) provide valid and reproducible results.

## Background

Severe yeast infections, especially candidaemia, represent a significant health problem in patients at high risk of infection, leading to increased morbidity and mortality, greater healthcare costs and increased duration of hospitalisation [1,2]. Although *Candida albicans *is the most common species associated with candidaemia, the incidence of non-*albicans Candida *spp. is increasing. According to a recent report from North America, there has been a change in the epidemiology of candidaemia in the US, with *C. albicans *responsible for only 45.6% of cases and *Candida glabrata *(26%) now the second most common cause of yeast infection [3], while in a previous report *C. albicans *was responsible for 58% of cases and *C. glabrata *for 20% of cases [4]. In Europe, *C. albicans *is still responsible for the majority (56.4%) of yeast infections; however, this figure is subject to considerable variation (42.7-67%) and is dependent on patient co-morbidities and risk factors [5]. In Italy, *Candida parapsilosis *is the second most common causative species, although it is less virulent than *C. glabrata *and *Candida tropicalis *[6-8]. Invasive infections caused by non *albicans Candida *spp. are more difficult to treat because of their innate or easily-acquired resistance to antifungal agents; therefore, the selection of drug treatment should be based on species-level identification. Furthermore, timely administration of antifungal drugs is mandatory because appropriate therapy and early treatment are associated with improved outcome in patients with fungal bloodstream infections [9].

The recent introduction of antifungal drugs with different mechanisms of action such as the echinocandins (e.g. caspofungin, micafungin and anidulafungin) and second-generation triazoles, together with the improved performance of antifungal susceptibility testing (AFST) methods justifies the greater use of AFST in clinical practice. Although AFST results should be interpreted with caution, particularly when commercial methods are used, they can serve as a useful guide in the selection of antifungal therapy. Clinical correlation between AFST results and patient outcome is very difficult to establish; however, it is widely accepted that a patient infected with a fungus with a high minimum inhibitory concentration (MIC) for a particular antifungal agent is unlikely to exhibit a good response to the drug [10]. Moreover national and local data on antifungal susceptibilities of clinically significant fungal strains are of interest to physicians, enabling them to adopt appropriate strategies for empiric and prophylactic therapies.

The present 2-year laboratory-based study was conducted to investigate the antifungal susceptibility of yeast species isolated from critically ill patients with invasive yeast infections in Italy in 13 laboratories centres (LC) and two co-ordinating centres (CC). The primary aim of the study was to characterize the freshly isolated yeast strains in terms of their *in vitro *susceptibility to systemic antifungal drugs that were available in Italy at the time of the study. As secondary objectives we evaluated the inter- and intra-laboratory reproducibility of two commercially available methods (Etest and Sensititre) for testing the susceptibility of yeasts to antifungal drugs and their level of agreement with the Clinical and Laboratory Standards Institute (CLSI) M27-A2 reference broth microdilution method [11].

## Methods

### Clinical isolates

All yeast isolates, recovered from the blood and sterile specimens of critically ill patients on general and surgical intensive care units and surgical wards over a period of 2 years (January 2007 to December 2008), were included in the study and analysed at 13 Italian local microbiology centres (LC). Isolates of the same species originating from the same patient were included only if they had been isolated from different specimens (i.e. blood and spinal fluid) or from the same specimen but at least 15 days apart. For this study, we did not use any additional data or samples other than those obtained during routine activity of our laboratories. Therefore, neither ethical approval nor patient consensus was considered necessary. The isolates were identified using standard procedures, (i.e. morphology on cornmeal agar plates, germ-tube production in serum, and biochemical analysis using the Vitek system Yeast Biochemical cards, API 20CAUX test, or ATB 32C panels [Bio-Merieux, Rome, Italy]) [12]. Prior to susceptibility testing, each isolate was sub-cultured on Sabouraud dextrose agar to ensure viability, purity, and optimal growth characteristics. After testing, each isolate was frozen at -80°C. Tested isolates were periodically transferred to one of two coordinating centres (CC) for the study, where they were re-tested and stored under appropriate conditions until the study was complete and the study analysis had been performed. In addition, the quality control isolates *Candida krusei *ATCC 6258 and *C. parapsilosis *ATCC 22019 listed in the CLSI M27-A2 document were tested [11].

### Drugs and reference method panels

The isolated yeast strains were tested for their *in vitro *susceptibility to amphotericin B, caspofungin, fluconazole, itraconazole, voriconazole, anidulafungin and posaconazole. Micafungin was not included because it was not available from the manufacturer at the time the study was conducted. Fluconazole, voriconazole and anidulafungin (Pfizer Pharmaceuticals, Groton, CT, USA), caspofungin (Merck & Co, Inc, Whitehouse Station, NJ, USA), and posaconazole (Schering Plough Corporation, Kenilworth, NJ, USA) were obtained as standard powders from their manufacturers; amphotericin B and itraconazole were purchased from Sigma-Aldrich, Milan, Italy. Caspofungin and fluconazole were dissolved in sterile water whereas all other drugs were dissolved in dimethyl sulfoxide. The broth microdilution panels for the reference method were prepared at each CC in accordance with the CLSI M27-A2 document [11] and literature data for the two echinocandins [13], stored at -80°C and used for testing within 6 months of the preparation date. With the exception of fluconazole (concentration range 0.125-128 mg/L), the antifungal drug concentrations ranged from 0.008 to 16 mg/L.

### Inoculum suspension

After overnight growth on Sabouraud dextrose agar at 35 °C, each isolate was suspended in 5 ml of sterile distilled water and thoroughly vortexed to achieve a smooth suspension. Turbidity (read at a wavelength of 530 nm) was adjusted to a McFarland standard of 0.5 with sterile distilled water. This suspension (approximately 1-5 x 10^6 ^CFU/mL) was used for Etest susceptibility testing (AB BIODISK, Solna, Sweden, now Bio-Merieux). For assays performed using broth microdilution methods, i.e. Sensititre (Trek Diagnostic Systems Ltd, East Grinstead, Sussex, UK) and the CLSI M27-A2 reference method, appropriate dilutions were prepared according to manufacturer recommendations or the standardized protocol (CLSI M27-A2) as appropriate.

### Susceptibility testing

All LCs and CCs performed susceptibility testing of each isolate using the Sensititre and Etest methods. In addition, the two CCs evaluated the antifungal susceptibility testing of the yeast isolates in comparison with the reference CLSI M27-A2 method.

Etest assays were performed using RPMI agar plates (Biolife, Milan, Italy), as recommended by the manufacturer. The plates were incubated at 35°C and read after 24 hours; if no growth was detected the plates were incubated for a further 24 hours. The drug concentration shown on the Etest strip at the outer border of the elliptical inhibition halo was recorded as the MIC. For triazoles, the growth of microcolonies within this inhibition zone was disregarded.

Panels for the Sensititre test were provided by Trek Diagnostic Systems. Readings were taken after 24 hours and if no growth was detected the panels were incubated for a further 24 hours. MICs were determined by visual inspection of the plates; the concentration of the first well that remained blue (absence of growth) was recorded as the MIC. For azoles, the bottom of each plate was visually inspected to avoid erroneous MIC readings caused by the trailing effect.

For the reference CLSI method, a visual reading was made after 24 hours of incubation, and the lowest concentration that produced a prominent decrease in turbidity ( ≥ 50%) relative to that of the drug-free control well was recorded as MIC. The MIC for amphotericin B was defined as the lowest concentration at which no visible growth was detected. After an additional incubation for 24 hours, the panels were analyzed spectrophotometrically (after shaking). For azoles and amphotericin B, the spectrophotometric reading (48 h) has been preferred to the recommended visual one to avoid bias related to the reader's expertise and used for the analysis [13].

### Data recording

Complete data (MIC values, date of the test, isolate number, related identifying number of the two quality control isolates tested in the same session) for each yeast isolate tested were recorded on an electronic data report form (E-DRF) by the investigator of each LC. The E-DRF was automatically checked by an electronic validation programme to verify the consistency of data. If the E-DRF passed this check, it was automatically saved, printed, and sent to the data management unit; otherwise, the user was prompted to add missing data. The printed copy of the E-DRF was signed by the investigator and filed on site. The same electronic and automated procedures were adopted for the CC results. After registration of the CC results, the CCs received a copy of the E-DRF containing the LC results where the yeast species had been isolated and tested.

### Analysis of results

The LC and CC MIC results were compared. For comparative purposes, the Etest MICs were adjusted to the nearest CLSI concentrations [14,15]. The MIC values were considered to be in essential agreement (EA) between two methods or two different tests when they were within 2 dilutions. Categorical agreement (CA) was assigned to Candida spp. results obtained by the two CC using the CLSI M27-A2 reference method and the two commercial methods that fell within the same interpretive categories (susceptible [S], non-susceptible [NS], susceptible dose-dependent [SDD] or resistant [R]) according to the following established CLSI MIC breakpoints for: fluconazole, S ≤8 mg/L, SDD 16-32 mg/L, R ≥64 mg/L; itraconazole S ≤0.125 mg/L, SDD 0.25-0.5 mg/L, R ≥1 mg/L; voriconazole S ≤1 mg/L, SDD 2 mg/L, R ≥4 mg/L [16]. The proposed clinical MIC breakpoints for anidulafungin and caspofungin used were S ≤2 mg/L, NS >2 mg/L [17]. Interpretive criteria for amphotericin B and posaconazole have not been established; instead, isolates inhibited by amphotericin B at concentrations ≤1 mg/L were considered susceptible and voriconazole breakpoints were applied to the posaconazole MIC values [18]. Discrepancies were considered "major" if an isolate classified as S by the reference method was classified as R by the commercial method and "very major" if an isolate classified as R by the reference method was classified as S by the commercial method. A minor error was recorded in the case of S vs SDD, R vs SDD, SDD vs S, or SDD vs R for the azoles. For echinocandins, categorical errors were considered "very major" when CLSI MIC indicated NS while Etest categorized the same isolate as S, and "major" when the Etest MIC indicated NS and CLSI S.

## Results

A total of 638 yeast isolates were collected from 578 patients during the study period. *C. albicans *(51.3%) was the most common species. *C. parapsilosis *(22.6%), *C. glabrata *(12.1%), *C. tropicalis *(6%), and *C. krusei *(2.4%) together with *C. albicans *represented 94.4% of all isolates tested. Non-*albicans Candida *represented 46.8% of the isolated species, including 7 *C. lusitaniae*, 7 *C. guilliermondii*, 4 *C. famata*, 3 *C. lipolytica*, 2 *C. sake *and 2 *C. utilis*. The remaining 1.9% belonged to other genera (10 *Cryptococcus neoformans*, and 1 each of *Geotrichum capitatum *and *Saccharomyces cerevisiae*). *C. parapsilosis *was more common in Southern and Central Italy than in Northern Italy (25.7% vs 19.9%), while the opposite was true for *C. glabrata *(7.5% vs 15.9%). Only the latter was statistically significant (p <0.001). Each of the 13 participating LC provided approximately 50 isolates.

The majority of the isolates were susceptible to all the antifungal drugs tested (Table 1). The overall MIC_90 _values as well as percentage of resistant isolates were influenced by the reduced susceptibility of *Candida parapsilosis *complex isolates to echinocandins and a reduced or lack of susceptibility of *Candida glabrata *and *Candida krusei *to azoles, in particular to fluconazole and itraconazole. For each drug, similar MIC_90 _values were generally obtained using Etest and Sensititre. The echinocandins were very active against *C. albicans*, *C. glabrata *and *C. krusei *isolates, although MIC_90 _values for anidulafungin were lower than those of caspofungin against the same species. High MIC values for both the echinocandins tested were determined for *C. guilliermondii*, *C. parapsilosis *complex, *Cryptococcus neoformans *and *Geotrichum capitatum*. With the exception of *C. glabrata *susceptibility to the azoles (the majority of MIC values were in the SDD or R category) and *C. krusei *susceptibility to fluconazole (MIC_90 _values were in the R category), all yeasts showed good susceptibility patterns to the azoles and amphotericin B. Overall, there was excellent categorical agreement between the reference method and the two commercial methods for all the antifungal drugs tested except for itraconazole. The CA percentage for itraconazole was very low for both the Etest and Sensititre methods; the majority of discrepancies were minor errors and were probably related to small differences in the MIC values for the itraconazole breakpoint (S ≤0.125 mg/L; SDD 0.25-0.5 mg/L; R ≥1 mg/L). The triazoles, fluconazole and posaconazole, exhibited poor agreement for *C. glabrata *with low CA percentage values (Etest: 44.2% and 51.9%, respectively; Sensititre: 40.3% and 68.8%, respectively), but these low CA values were predominantly associated with minor errors. The CA percentage for fluconazole was also low for *C. krusei *(Etest: 46.7%; Sensititre: 33.3%), in this case a high number of very major errors occurred with Sensititre method.

**Table 1:** In vitro antifungal susceptibilities of 638 clinical yeast isolates determined by CLSI M27-A2, Etest and Sensititre

Drug	Method	MIC values			% of R strains	CA%	% of isolates with discrepant results		
					
		Ranges	50%	90%			Minor	Major	Very major
	CLSI	0.12-2	0.5	1	2.6				
Amphotericin B	Etest	0.032-8	0.19	0.38	0.2	97.5	-	-	2.5
	Sensititre	0.03-2	0.5	1	0.4	97.3	-	0.2	2.5

	CLSI	<0.008-≥16	0.12	16	3.6				
Anidulafungin	Etest	≤0.002-≥32	0.008	1.5	3.8	94.7	-	2.1	3.2
	Sensititre	≤0.008-≥16	0.06	1	1.7	96.4	-	-	3.6

	CLSI	0.016-≥16	1	2	6.7				
Caspofungin	Etest	0.012-≥32	0.19	0.75	2.2	95.0	-	0.2	4.8
	Sensititre	0.015-≥16	0.12	0.5	2.2	95.3	-	-	4.7

	CLSI	≤0.12-128	0.5	16	3.3				
Fluconazole	Etest	0.032-≥256	0.38	8	2.2	91.1	7.7	0.2	1
	Sensititre	≤0.12-≥256	0.5	16	2.7	89.8	0.2	0.2	9.8

Itraconazole	CLSI	0.008-≥16	0.25	1	7.8				
	Etest	0.004-≥32	0.064	1.5	8.9	47.7	49.2	-	3.1
	Sensititre	≤0.008-≥8	0.03	1	5.9	46.8	59.2	-	4.0

Posaconazole	CLSI	≤0.008-≥8	0.12	1	6.5				
	Etest	0.003-≥32	0.064	0.5	10.6	93.4	4.8	1.6	0.2
	Sensititre	≤0.008-≥8	0.03	1	2.3	96.0	3.5	-	0.5

Voriconazole	CLSI	≤0.008-8	0.016	0.25	1.9				
	Etest	≤0.002-≥32	0.023	0.25	1.4	98.8	0.9	-	0.3
	Sensititre	≤0.008-8	0.008	0.25	1.1	98.3	1.0	0.2	0.5

* Categorical Agreement (MIC values that fell within the same interpretive categories)

**Figure 1:**
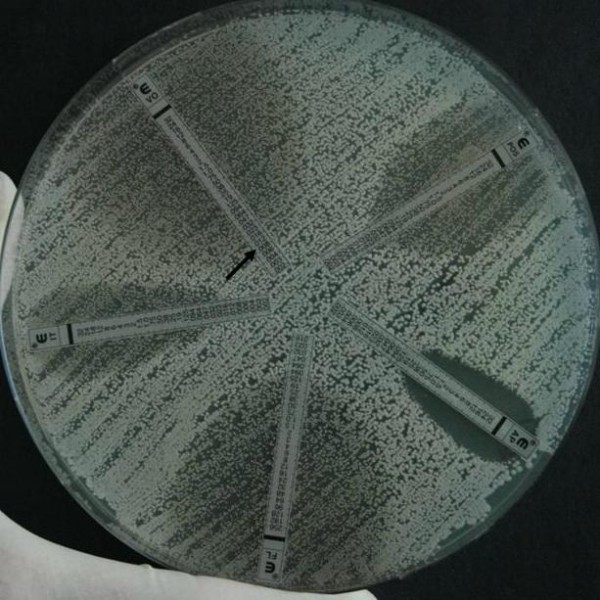
**Etest results of a *Candida albicans *clinical isolate tested against amphotericin B, fluconazole, itraconazole, posaconazole, and voriconazole**. Note the lawn of microcolonies inside the ellipses of triazole strips; according to the endpoint rule recommended by the manufacturer, the minimum inhibitory concentration for voriconazole should be 0.008 mg/L, i.e. the first change in growth (black arrow).

Comparison of the LC and CC results showed good EA (90.3% for Etest and 92.9% for Sensititre), and even higher CA (93.9% for Etest and 96% for Sensititre); differences were observed according to the species, method, and antifungal drug (Table 2). The overall EA for both echinocandins, anidulafungin and caspofungin, was high with both methods, although a slightly better agreement was observed for the Sensititre results. However, this difference was not evident for the CA, although differences were observed among the single species. As expected, *C. parapsilosis *isolates were generally associated with high echinocandin MIC values, with several isolates above the CLSI breakpoint [17]; this was more evident for the Etest than for the Sensititre results. In general, EA percentage for the azoles was lower with the Etest than with the Sensititre method. This confirmed in part the bias associated with subjective reading of the Etest results, which for some species (e.g. *C. albicans *and *C. tropicalis*) is complicated by the presence of a double halo or lawn of microcolonies within the discernible ellipse (Figure 1). Itraconazole showed a very low agreement between LC and CC results with both methods, in particular for *C. glabrata, C. tropicalis *and *C.krusei *isolates. *C. glabrata *was the most problematic species for the azoles; EA was <70% for itraconazole and posaconazole using the Etest, and the CA percentage was very low for fluconazole, itraconazole, and posaconazole with both methods (Etest 64.9%, 80.3%, 57.1%, respectively; Sensititre 58.4%, 66.2%, 77.9%, respectively).

**Table 2:** Comparison between LC* and CC* in vitro antifungal susceptibilities of the most representative *Candida *species determined by Etest and Sensititre

Drug	Species (No of isolates)	Centre	Etest MIC mg/L			°Agreement %		Sensititre MIC mg/L			°Agreement %	
			Range	50%	90%	EA	CA	Range	50%	90%	EA	CA
	All isolates (638)	LC	≤0.002-1.5	0.19	0.5	93.1	99.4	0.015-2	0.5	1	89.9	98.6
		CC	0.032-8	0.19	0.38			0.03-2	0.5	1		
	
	*Candida albicans *(327)	LC	0.002-1	0.19	0.25	96.3	100	0.015-1	0.5	1	91.1	99.4
		CC	0.032-1	0.19	0.25			0.06-1	0.5	0.5		
	
	*Candida parapsilosis *(144)	LC	0.004-1.5	0.125	0.5	85.4	99.3	0.015-2	0.5	1	89.6	98.6
Amphotericin B		CC	0.032-0.75	0.19	0.25			0.03-1	0.5	0.5		
	
	*Candida glabrata *(77)	LC	0.002-1.5	0.25	0.75	97.4	98.7	0.015-2	0.5	1	87	98.7
		CC	0.047-1	0.25	0.5			0.06-1	0.5	1		
	
	*Candida tropicalis *(38)	LC	0.032-1.5	0.25	0.5	86.8	97.4	0.06-2	0.5	2	97.7	89.5
		CC	0.047-0.75	0.25	0.5			0.12-1	0.5	1		
	
	*Candida krusei *(15)	LC	0.064-0.5	0.5	0.5	93.3	100	0.25-1	0.5	1	100	93.2
		CC	0.25-1	0.5	1			0.5-2	1	1		

	All isolates (638)	LC	≤0.002-≥32	0.008	2	89.6	93	≤0.008-≥16	0.06	1	92.5	99.7
		CC	≤0.002-≥32	0.008	1.5			≤0.008-≥16	0.06	1		
	
	*Candida albicans *(327)	LC	≤0.002-4	0.003	0.006	89.7	99	≤0.008-1	0.03	0.06	89.6	100
		CC	≤0.002-3	0.003	0.006			≤0.008-2	0.03	0.06		
	
Anidulafungin	*Candida parapsilosis *(144)	LC	0.004-≥32	1.5	4	89.2	74.1	0.03-4	1	2	97.9	98.6
		CC	≤0.002-≥32	1	2			≤0.008-2	1	2		
	
	*Candida glabrata *(77)	LC	0.003-2	0.012	0.032	92.0	100	≤0.008-2	0.03	0.12	94.8	100
		CC	0.004-2	0.012	0.023			≤0.015-2	0.06	0.12		
	
	*Candida tropicalis *(38)	LC	≤0.002-0.064	0.016	0.032	84.2	100	≤0.008-1	0.06	0.25	89.5	100
		CC	0.006-1.5	0.016	0.125			0.03-2	0.06	0.25		
	
	*Candida krusei *(15)	LC	0.012-0.047	0.023	0.047	100	100	0.015-0.12	0.06	0.12	100	100
		CC	0.012-0.047	0.023	0.047			0.06-0.12	0.06	0.12		

	All isolates (638)	LC	≤0.002-≥32	0.19	1	94.5	98.9	≤0.008-≥16	0.12	0.5	96.7	99.8
		CC	0.012-≥32	0.19	0.75			0.015-≥16	0.12	0.5		
	
	*Candida albicans *(327)	LC	≤0.002-1	0.125	0.25	94.2	100	0.008-0.5	0.06	0.12	97.9	100
		CC	0.012-1.5	0.094	0.19			0.015-1	0.06	0.12		
	
	*Candida parapsilosis *(144)	LC	0.032-3	0.75	2	92.3	95.8	0.03-8	0.5	1	95.8	99.3
Caspofungin		CC	0.094-3	0.5	1			0.06-2	0.5	1		
	
	*Candida glabrata *(77)	LC	0.047-2	0.25	0.38	98.7	100	0.015-≥16	0.12	0.25	97.4	100
		CC	0.125-1	0.19	0.25			0.03-≥16	0.12	0.25		
	
	*Candida tropicalis *(38)	LC	0.016-0.38	0.19	0.25	94.7	100	0.03-0.5	0.06	0.25	92.1	100
		CC	0.064-2	0.19	0.5			0.03-0.5	0.06	0.25		
	
	*Candida krusei *(15)	LC	0.38-1	0.75	1	100	100	0.12-1	0.5	0.5	100	100
		CC	0.38-1.5	0.5	1			0.12-0.5	0.5	0.5		

	All isolates (638)	LC	0.023-≥256	0.38	12	92.2	93.2	≤0.12-≥256	0.5	16	94.3	92.8
		CC	0.032-≥256	0.38	8			≤0.12-≥256	0.5	16		
	
	*Candida albicans *(327)	LC	0.023-≥256	0.25	0.75	92.0	99.1	≤0.12-≥256	0.25	0.5	94.8	99.1
		CC	0.047-32	0.25	0.75			≤0.12-32	0.25	0.5		
	
	*Candida parapsilosis *(144)	LC	0.32-≥256	0.5	2	95.8	97.2	≤0.12-128	1	4	97.2	96.7
Fluconazole		CC	0.125-32	0.5	1.5			≤0.12-64	1	4		
	
	*Candida glabrata *(77)	LC	0.38-≥256	12	≥256	84.4	64.9	0.5-≥256	16	64	90.9	58.4
		CC	0.125-≥256	8	48			0.5-≥256	16	64		
	
	*Candida tropicalis *(38)	LC	0.125-16	0.5	2	92.1	100	0.25-≥256	1	4	84.2	92.1
		CC	0.032-32	0.5	2			0.25-16	1	4		
	
	*Candida krusei *(15)	LC	12-≥256	48	≥256	86.7	46.7	8-64	32	64	100	86.7
		CC	16-128	32	64			32-64	32	64		

	All isolates (638)	LC	≤0.002-≥32	0.064	1	81.3	79.4	≤0.008-≥16	0.06	0.5	92.5	84.4
		CC	0.004-≥32	0.064	1.5			≤0.008-≥16	0.06	0.5		
	
	*Candida albicans *(327)	LC	0.002-≥32	0.047	0.25	84.1	80.4	≤0.008-≥16	0.03	0.12	92.7	94.1
		CC	0.004-1	0.064	0.25			≤0.008-0.25	0.06	0.12		
	
	*Candida parapsilosis *(144)	LC	≤0.002-1	0.032	0.25	87.5	84.7	≤0.008-0.5	0.12	0.12	94.4	82.7
Itraconazole		CC	0.008-3	0.032	0.38			≤0.008-1	0.12	0.25		
	
	*Candida glabrata *(77)	LC	≤0.002-≥32	1.5	≥32	61.8	80.3	0.03-≥16	0.5	≥16	89.6	66.2
		CC	0.004-≥32	2	≥32			0.06-≥16	0.5	2		
	
	*Candida tropicalis *(38)	LC	0.012-16	0.094	0.38	68.4	55.3	0.03-≥16	0.12	0.5	86.8	36.8
		CC	0.016-3	0.125	1			0.06-1	0.25	0.5		
	
	*Candida krusei *(15)	LC	0.125-32	1	2	86.7	73.3	0.12-0.5	0.25	0.5	100	73.3
		CC	0.5-2	1	2			0.12-0.5	0.25	0.5		

	All isolates (638)	LC	≤0.002-≥32	0.064	0.5	92.8	94.2	≤0.008-≥8	0.03	0.5	91.8	96.1
		CC	0.003-≥32	0.064	0.5			≤0.008-≥8	0.03	1		
	
	*Candida albicans *(327)	LC	≤0.002-≥32	0.047	0.094	95.7	99.7	≤0.008-≥8	0.015	0.03	90.8	98.8
		CC	0.003-0.25	0.047	0.094			≤0.008-0.5	0.015	0.03		
	
	*Candida parapsilosis *(144)	LC	0.003-0.25	0.064	0.125	98.6	99.3	≤0.008-4	0.03	0.12	95.1	99.3
Posaconazole		CC	0.012-4	0.047	0.125			≤0.008-1	0.06	0.12		
	
	*Candida glabrata *(77)	LC	0.003-≥32	1	≥32	66.2	57.1	≤0.008-≥8	0.5	≥8	90.9	77.9
		CC	0.012-≥32	1	24			0.015-≥8	1	≥8		
	
	*Candida tropicalis *(38)	LC	0.016-1.5	0.094	0.19	94.7	97.4	0.015-≥8	0.12	1	84.2	92.1
		CC	0.016-2	0.094	0.25			0.015-1	0.12	1		
	
	*Candida krusei *(15)	LC	0.094-1	0.25	0.5	93.3	100	0.12-0.5	0.25	0.5	100	100
		CC	0.19-0.5	0.25	0.5			0.12-0.5	0.25	0.5		

	All isolates (638)	LC	≤0.002-≥32	0.016	0.25	90.3	98.9	≤0.008-≥16	0.008	0.25	91.5	99.0
		CC	≤0.002-≥32	0.023	0.25			≤0.008-8	0.008	0.25		
	
	*Candida albicans *(327)	LC	≤0.002-≥32	0.012	0.032	86.2	99.4	≤0.008-≥16	≤0.008	0.015	92.7	99.4
		CC	≤0.002-0.25	0.016	0.047			≤0.008-0.25	≤0.008	≤0.008		
	
	*Candida parapsilosis *(144)	LC	≤0.002-1	0.016	0.094	91.7	100	≤0.008-1	0.015	0.12	90.3	100
Voriconazole		CC	≤0.002-1	0.016	0.094			≤0.008-1	0.015	0.06		
	
	*Candida glabrata *(77)	LC	0.004-≥32	0.25	1.5	85.7	94.8	≤0.008-≥16	0.25	1	89.6	89.6
		CC	0.006-≥32	0.25	1.5			≤0.008-8	0.25	1		
	
	*Candida tropicalis *(38)	LC	0.016-0.5	0.047	0.125	86.8	100	≤0.008-16	0.06	1	86.8	94.7
		CC	0.012-1	0.064	0.25			0.015-1	0.06	0.25		
	
	*Candida krusei *(15)	LC	0.094-0.75	0.25	0.5	100	100	0.06-0.5	0.25	0.5	100	100
		CC	0.19-0.75	0.38	0.5			0.12-0.5	0.25	0.5		

* LC = Laboratory Centre, CC = Coordinating Centre

° EA = Essential Agreement (MIC values within 2 dilutions), CA = Categorical Agreement (MIC values that fell within the same interpretive categories),

## Discussion

The regional variation in the distribution of various *Candida *species observed in this study, in particular differences in the occurrence of *C. glabrata *and *C. parapsilosis *in northern versus central/southern Italy, may be attributable to differences relating to climate, the management of vascular catheters, and the empirical and prophylactic use of antifungal drugs [7,8].

Our data on antifungal susceptibility suggest that antifungal resistance is low among yeasts isolated from critically ill patients with invasive infections in Italy. These results agree with those reported in other Italian publications, although the studies were conducted on a regional rather than a national basis [2,19-21]. Furthermore, our findings highlight the importance of adopting current clinical practice guidelines for the management of invasive yeast infections, particularly candidiasis; these include European Guidelines for the Management of Bacterial and Fungal Infections developed by the European Conference on Infections in Leukaemia (ECIL) [22] and by the Infectious Diseases Society of America (IDSA) [2].

Resistance to the recently introduced echinocandins was reported very rarely in our study; only *C. neoformans *isolates were resistant to both echinocandins, and the single strain of *Geotrichum capitatum *showed high MIC values for both anidulafungin (8 mg/mL) and caspofungin (≥16 mg/L). Some isolates of the *C. parapsilosis *complex and *C. guilliermondii *demonstrated a decreased susceptibility to these drugs, particularly anidulafungin which had Etest MIC values that were higher than the proposed breakpoint [17]. Further support for these findings was provided by our comparison of the *C. parapsilosis *complex Etest results with those of the reference method at the CCs which revealed a higher percentage CA between the reference and Sensititre methods than between the reference and Etest methods. Although we detected a lower percentage of very major (6.2% of false susceptibility) errors compared with the 14% detected by Espinel-Ingroff and coworkers [23], this observation agrees with their data and corroborates their conclusion that the Etest is not suitable for testing the susceptibility of anidulafungin against *C. parapsilosis *complex [23].

The majority of our isolates were susceptible *in vitro *to amphotericin B and the triazoles, although differences were observed across species. As expected, *C. krusei *demonstrated innate resistance to fluconazole but was susceptible to posaconazole and voriconazole with MIC values of 0.5 mg/L for 100% of the isolates tested. The majority of *C. glabrata *isolates were not susceptible (SDD or R category) to triazoles, although differences were observed for individual triazoles which were probably related to the different efflux pumps involved in the development of *C. glabrata *azole resistance [24]. Voriconazole was the most active azole compound against *C. glabrata *with a few isolates exhibiting resistance; this is in contrast to other reports suggesting that isolates classified as fluconazole resistant are also resistant to voriconazole [25]. One major problem we encountered with *C. glabrata *was the low rate of agreement between the two commercial susceptibility test methods and the reference method, especially for fluconazole and posaconazole. However, this is in agreement with earlier studies that we have conducted comparing commercial methods with the CLSI reference method [26,27]. The poor performance of itraconazole could be attributable to the small differences in MIC values for the itraconazole breakpoint.

## Conclusions

Our study demonstrates that clinical yeasts, isolated from blood and sterile specimens, are generally susceptible to currently available antifungal drugs indicated for the treatment of invasive yeast infections in Italy. However, identification of the yeast species and strain together with antifungal susceptibility testing of clinically relevant yeasts is essential to achieve optimal clinical outcome. Selection of the most appropriate method for susceptibility testing can be problematic as the low percentages of CA and EA Etest demonstrated for some *Candida *species. However, with the exception of these few species (e.g. *C. glabrata *for azoles and *C. parapsilosis *for echinocandins), the findings of our study suggest that two of the most widely used commercial methods (Etest and Sensititre) provide valid and reproducible results as demonstrated by the blind susceptibility re-testing of all yeast isolates at the study CCs. Caution should be adopted when elevated MIC values are determined by these commercial methods among isolates of *Candida *species generally susceptible to a given antifungal drug; the possible resistance should be confirmed by reference methods.

## Competing interests

GM has acted as a consultant for Astellas Pharma SpA, MSD Italia and Pfizer Italia, received honoraria from Astellas Pharma SpA, MSD Italia, Pfizer Italia and Schering Plough Italia, and received a grant from Pfizer Italia.

MTM has received honoraria from MSD Italia, Pfizer Italia, Schering Plough Italia and Gilead, and received a grant from Pfizer Italia.

EB, RI, GA, SA, GB, CF, GLC, GL, EM, MM, PP, RR, ET, MV have no conflict of interest to declare

## Authors' contributions

GM and MTM designed and co-ordinated the study, analyzed and interpreted the data, and drafted the manuscript; EB and RI carried out all tests at the two co-ordinating centres; GA, SA, GB, CF, GLC, GL, EM, MM, PP, RR, ET, MV participated in the design of the study, performed the tests at the laboratory centres, and collected the isolates. All authors read and approved the final manuscript.

## Pre-publication history

The pre-publication history for this paper can be accessed here:

http://www.biomedcentral.com/1471-2334/11/130/prepub
